# A review of the nutritional value and biological activities of sturgeon processed byproducts

**DOI:** 10.3389/fnut.2022.1024309

**Published:** 2022-11-14

**Authors:** Rui Chen, Zhe Liu, Jinze Wang, Wengang Jin, Hassan Idris Abdu, Jinjin Pei, Qi Wang, A. M. Abd El-Aty

**Affiliations:** ^1^Shaanxi Province Key Laboratory of Bioresources, 2011 QinLing-Bashan Mountains Bioresources Comprehensive Development C. I. C., Qinba State Key Laboratory of Biological Resources and Ecological Environment, College of Bioscience and Bioengineering, Shaanxi University of Technology, Hanzhong, China; ^2^State Key Laboratory of Biobased Material and Green Papermaking, Qilu University of Technology, Shandong Academy of Sciences, Jinan, China; ^3^Department of Pharmacology, Faculty of Veterinary Medicine, Cairo University, Giza, Egypt; ^4^Department of Medical Pharmacology, Faculty of Medicine, Atatürk University, Erzurum, Turkey

**Keywords:** sturgeon, nutrients, byproducts, processing, biological activities

## Abstract

Sturgeons are a type of subcold water fish distributed in eastern Europe, on both sides of the North Pacific, in eastern Asia, in western North America, and on the east coast of North America. Its production capacity is strong, and it is easy to breed. However, the sturgeon industry has the problems of a single product structure, a short industrial chain and poor market sales. In this context, developing the sturgeon industry is crucial to research the nutritional value of sturgeon processing byproducts and developing diversified products. Therefore, this paper summarizes the research on the nutritional value of sturgeon processing byproducts and the current situation of processing and utilization over the past 10 years. First, CiteSpace visual analysis software and the bibliometric analysis platform were used to analyze the status of sturgeon research. The Web of Science (WOS) database was used as the literature source to fit the keywords of sturgeon literature in the past ten years. After excluding the two keywords sturgeon and sturgeon meat, the relevant literature is analyzed and sorted, focusing on the literature in the last five years. Second, a comprehensive and in-depth review (sturgeon, processing, byproducts as the keywords to search Google Scholar and Web of Science) was conducted on the research of the nutritional components contained in sturgeon and the processing of nutritional components in byproducts to provide a reliable reference for the research and processing of the sturgeon industry.

## Introduction

*Acipenser sturio Linnaeus* is one of the oldest fishes in the world, including two families and 27 species belonging to the class teleost, the subclass radiofin, the order sclerolepidoptera and the order sturgeon (1–3). Acipenser sturgeon is a subcold water fish, mainly distributed on the east coast of the Pacific Ocean, the Great Lakes region of North America, the northwest of the Atlantic Ocean, the Mississippi River basin and the Gulf of Mexico in North America, the northeast of the Atlantic Ocean, the Caspian Sea region, Siberia and the Arctic Ocean basin, Heilongjiang and the Sea of Japan, the Yangtze River and the Pearl River. Sturgeon meat is delicious and rich in nutrients, and its byproducts are also popular. For example, caviar made from sturgeon eggs is famous. People call caviar, goose liver and truffle the three top delicacies in the world. Since China first exported sturgeon caviar in 2006, the annual export volume has increased yearly. Sturgeon not only has high nutritional value but also has very high medicinal and ornamental value. Sturgeon is rich in eight essential amino acids and has a higher crude protein content than pork, eggs, river crabs, carp, crucian carp, and bighead carp. The crude fat content is higher than shrimp and tilapia. Sturgeon muscle contains extremely low zinc, phosphorus, magnesium, iron, potassium, sodium, calcium, manganese and copper contents. Among them, the contents of potassium and phosphorus are the highest and are higher than those of pork, beef, chicken, eggs, prawns, river crabs, and common fish, such as green, grass, silver carp, and bighead carp (4–6). Regarding the medicinal value, the nutrient composition in sturgeon cartilage is comparable to that of sharks, and the rich anticancer factor (bioactive active ingredient) is 15–20 times that of sharks (7–9). Sturgeon gills have a remarkable effect on heat clearing and detoxification. Sturgeon oil has a wonderful effect on scalds. Sturgeon is rich in “DHA” and “EPA,” called brain gold. It has a good effect on softening the heart and brain vessels, promoting brain development, improving IQ, and preventing senile dementia (10). With the gradual maturity of artificial culture and seedling breeding technology for sturgeon, the production of sturgeon has increased rapidly. However, because sturgeon is not a widely consumed aquatic product, with a large number of commercial fish coming on the market, the market price has fallen rapidly. In just a few years, the average price of commercial fish has dropped from the highest 600 RMB/kg to the current 60 RMB/kg, a decrease of 90% (2, 3, 5, 11). With the rise of comprehensive breeding costs, overcapacity and falling prices, the development of the sturgeon industry gradually slows down.

**FIGURE 1:**
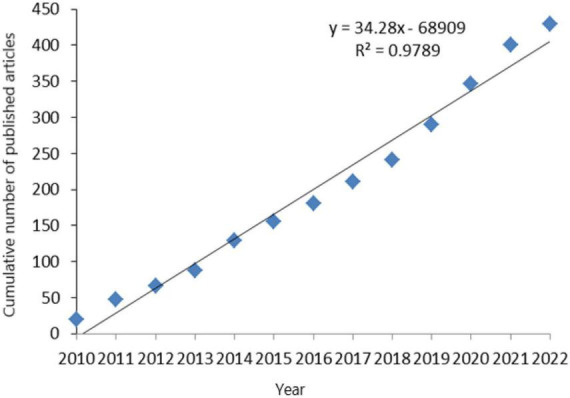
Regression verification of the annual document volume of the WOS database.

**FIGURE 2:**
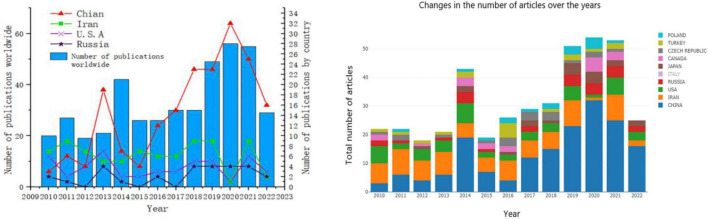
Analysis of global document issuance from 2010 to 2022.

Based on the retrieval and analyses of the sturgeon’s number of documents, it is found that the number of documents issued for the sturgeon has increased linearly from 2010 to 2022. The cumulative number of documents issued for the sturgeon has maintained stable growth over the past 10 years, indicating that research on the sturgeon has always maintained a stable state (Figure 1). Through the statistics of the number of documents issued by different countries, it is found that, as shown on the right of Figure 2, the top ten countries in the world in terms of the number of documents issued during this decade are China, Iran, the United States, Italy, Russia, Japan, Canada, the Czech Republic, Türkiye and Poland, of which China has 198 documents, far exceeding Iran (82) and the United States (49). As shown on the left of Figure 2, from 2010 to 2018, the number of documents issued by sturgeon deep processing remained constant. From 2019 to 2022, the number of documents issued by sturgeon deep processing ushered in a leap, and in 2014, the number of documents issued by sturgeon deep processing ushered in a sudden increase. China caused changes in the number of documents issued in both cases. In the past 12 years, Chinese sturgeon processing has developed rapidly. In 2013, China surpassed Iran and the United States to become the country with the largest number of documents on sturgeon deep processing. In the following years, the number of documents issued rose sharply, making outstanding contributions to sturgeon processing research. This is related to China being the first aquaculture country and the first exporter of sturgeon. However, in recent years, sturgeon market sales have been poor, and the market situation is overcapacity (4, 11). Therefore, this paper analyzes the nutrient composition and global research status of sturgeon, intending to increase the added value of sturgeon and improve the sturgeon market.

## Nutritional components and biological activities of sturgeon byproducts

### Proteins and active peptides in sturgeon byproducts

Protein is the most crucial nutrient for the human body. Insufficient protein nutrition will lead to stunting, anemia, weakness, edema, vascular dysfunction, and impaired immune function. Protease and peptidase degrade and produce amino acids, dipeptides, and tripeptides in the lumen of the gastrointestinal tract, which is then absorbed by the human body. Essential amino acids are the amino acids that the human body cannot synthesize, or the synthesis speed cannot meet the needs of the human body and must be taken from food. Therefore, it is essential to take enough high-quality protein from animal products for the optimal growth, development, and health of human beings (12). Sturgeon products are rich in essential amino acids. In recent years, an increasing number of people have studied the protein nutritional components of sturgeon. Gao Lujiao found that the average content of crude eggs in the eggs of Russian sturgeon and Siberian sturgeon is approximately 20.38–20.77%, and the eggs of both species contain 17 different amino acids (13). Zhao Zhongmeng analyzed the nutritional components of Siberian sturgeon, Amur sturgeon Schrenckii and their hybrids and scored their proteins using the FAO/WHO amino acid scoring model. The study showed that the ratios of essential amino acids to total amino acids of the three sturgeon species were 40.70, 41.24, and 40.08%, and the ratios of essential amino acids to non-essential amino acids were 68.75, 70.20, and 66.84%. Compared with the ideal FAO/WHO model, the ratio of essential amino acids to total amino acids was 40%. The ratio of essential amino acids to non-essential amino acids is more than 60%, which is a good protein, so the proteins of the three sturgeons all meet the requirements of high-quality protein. Moreover, the highest lysine content of the three sturgeon species exceeded the FAO/WHO model and egg protein, and lysine is known as a “growth amino acid” (14). Huangpan et al. studied a hybrid sturgeon (*Acipenser schrenckii* × *Huso dauricus*). It was found that the ratio of essential amino acids to total amino acids in all parts of this hybrid sturgeon was 45–46%, higher than 40% of the ideal model of FAO/WHO, indicating that its protein is a high-quality protein (15). Chen Yuewen divided the Russian sturgeon into eight regions from head to tail and analyzed its protein. The research showed that the ratio of essential amino acids to total amino acids in the Russian sturgeon was 39.87–43.09%, with an average of 42.36%. The ratio of essential amino acids to non-essential amino acids was 79.61–91.73%, much higher than 60%. It also proved that sturgeon protein was high-quality (16). Anti-inflammatory and antioxidant peptides have been widely studied in recent years because they can act as effective adjuvants, cooperate with other immune factors, strengthen the adaptive response, and promote wound healing (17). Zhang et al. found that the intron in the antibacterial peptide gene of sturgeon is much longer than that of other organisms and has a pronounced response effect to *Aeromonas hydrophila* (18). The polypeptide isolated from sturgeon skin gelatin by Nikoo et al. can scavenge DPPH, ABTS and hydroxyl radicals and inhibit fat oxidation of COD minced fish (19).

### Mineral elements in sturgeon byproducts

Mineral elements are nutritional elements that cannot be synthesized in the body and can only be obtained from the outside world. However, when the daily intake of trace elements is insufficient, it will impact human health, such as increased susceptibility to infection, resulting in fatigue, lack of energy and inattention (20). Research has shown that sturgeon bones and meat contain rich types of trace elements required by the human body. Shixiaoling et al. studied the content of trace elements in sturgeon cartilage in the Yangtze River Basin. They found that the contents of Fe, Cu, Zn, and other trace elements are rich, while the content of Mn is relatively low (21). Ca is not only an essential material for the growth and development of human bones but also an element necessary for maintaining and regulating the normal physiological functions of nerves, muscles, blood, cell membranes and the activities of various enzymes. Mg is a cation in cells, mainly concentrated in mitochondria, and plays a role in many enzyme systems, especially the biological activities of enzyme systems related to oxidative phosphorylation; Fe has a hematopoietic function in the human body, participates in the synthesis of blood protein, cytochrome, and various enzymes, and promotes human growth; Cu and Zn are essential elements for animal growth. After being absorbed, most of them participate in synthesizing biological enzymes *in vivo* (20). Huang et al. also showed that these trace elements are detected in sturgeon year round. Among them, Mg, K, Mn, and Fe are the highest in spring, while Na, Ca, and Zn are the highest in summer. Therefore, in addition to direct cooking, sturgeon processing wastes such as sturgeon bone can also be used as high-quality raw materials for other processed products (22).

### Other bioactive substances in sturgeon byproducts

Sturgeon is also rich in other bioactive substances. Zhouwanjun et al. analyzed chondroitin sulfate from Siberian sturgeon. They found that chondroitin sulfate from Siberian sturgeon has a scavenging effect on DPPH hydroxyl radicals and OH, and the clearer it is with the increase in the sulfate cartilage concentration (23). Huangshiyu found that sturgeon chondroitin sulfate can significantly improve the immune function and anti-inflammatory and anti-allergic activities of mice (24). Karimzadeh pointed out that glycosaminoglycans extracted from sturgeon bones have good angiotensin-converting enzyme inhibition and anticoagulation effects and can replace blood-lowering drugs and antithrombotic drugs under laboratory conditions (17). In addition, fatty acids are important bioactive substances. Many reports have shown that specific fatty acids have beneficial effects on human health, which may help prevent many chronic diseases in humans. A considerable amount of evidence has accumulated to support the view that the very long chain omega 3 fatty acids [eicosapentaenoic acid (EPA) and docosahexaenoic acid (DHA)] have beneficial cardiovascular and anti-inflammatory properties and that levels of their consumption are insufficient in most Western diets (25). However, a large number of studies have shown that sturgeon is rich in EPA and DHA, two bioactive substances. Chen Yuewen’s (16) research shows that the flesh of Russian sturgeon contains 22 fatty acids, among which oleic acid has the highest content. Oleic acid regulates blood glucose and lipids and lowers cholesterol. In addition, sturgeon also has a large amount of palmitic acid (C16:0, 18.15–21.21%) and linoleic acid (C18:2n6c, 23.20–28.31%). The total content of EPA and DHA, which are of concern to people, is 3.37–4.26%, which is significantly higher than that of crucian carp and tilapia. Huang et al. studied the hybrid sturgeons of Amur sturgeon Acipenser schrenckii and Huso dauricus and found that palmitic acid (C16:0, 18.89–21.02%) was the main SFA in all parts; oleic acid (C18:1, 31.81–34.08%) was the main MUFA; and in PUFA, the content of linoleic acid (C18:2, 17.72–22.34%) was the highest, followed by DHA (C22:6, 4.56–6.14%) and EPA (C20:5, 3.13–3.57%) (15). The contents of palmitic acid, oleic acid, linoleic acid, DHA, and EPA in Siberian sturgeon reported by Nieminen (26) and Pyz Lukasik (27) are also similar to those reported by Huang and Chen.

## Literature analysis of sturgeon research

CiteSpace is a visual analysis software developed by Dr. Chenchaomei of Redsell University in the United States, which can be used to analyze the potential multivariate, time-sharing, dynamic and other knowledge in the scientific literature (28–30).

This article uses “sturgeon” as the keyword to search the relevant literature from 2010 to 2022 on the WOS platform. There were 4555 papers, 426 conference papers and 149 review papers. A total of 433 papers related to sturgeon deep processing were obtained by screening the retrieved papers. The selected literature was exported and preserved, and then the authors and keywords were studied and clustered with the help of the CiteSpace software developed (31, 32). Through the WOS platform^1^ and the bibliometric analysis platform^2^ (33), we drew and analyzed the cooperation relationship and the number of documents issued in the sturgeon research field (34–37).

### Analysis of research country and issuing agency

Table 1 is obtained by analyzing the literature of major research institutions in the field of sturgeon deep processing in the world from 2010 to 2022. According to the ranking of papers published in Table 1, the research institution with the most papers published is the Chinese Academy of Fishery Sciences, followed by Islamic Azad University and Jiangsu University. Among the top 10 institutions, 6 were in China, 2 were in Iran, 1 was in Japan, and 1 was in the Czech Republic. China accounts for 60% of these institutions, but the United States and Russia, which account for the top four in the number of papers, are not among the top 10 research institutions. According to the ranking of total citations in Table 1, among the top 10 research institutions, there are 7 in China, 1 in Iran and 1 in Japan. China accounts for a very high proportion in terms of the number of documents issued and the total number of cited schools, indicating that China has the largest volume of sturgeon deep processing research worldwide. Among them, Jiangsu University, Jiangnan University, China Agricultural University and Huazhong Agricultural University are famous universities in the field of food. The two institutions with the highest average number of visits are Jiangnan University and China Agricultural University, key research institutions in the global food field.

**TABLE 1:** Major research institutions in the field of sturgeon deep processing in the world from 2010 to 2022.

Ranking of published papers	Institution	Number of articles	Total cited times	Average cited times	Total cited ranking	Institution	Number of articles	Total cited times	Average cited times
1	Chinese Acad Fishery Sci	52	88	1.69	1	WSCS	5	134	26.80
2	Islamic Azad Univ	32	32	1.00	2	Chinese Acad Fishery Sci	52	88	1.69
3	Jiangsu Univ	31	65	2.10	3	Jiangsu Univ	31	65	2.10
4	Hokkaido Univ	29	65	2.24	4	Hokkaido Univ	29	65	2.24
5	China Agr Univ	29	35	1.21	5	Zhejiang Gong shang Univ	25	59	2.36
6	Zhejiang Gong shang Univ	25	59	2.36	6	Jiangnan Univ	13	49	3.77
7	Dalian Polytech Univ	24	37	1.54	7	Huazhong Agr Univ	11	44	4.00
8	Tarbiat Modares Univ	17	31	1.82	8	Dalian Polytech Univ	24	37	1.54
9	Univ South Bohemia Ceske Budejovice	14	11	0.79	9	China Agr Univ	29	35	1.21
10	Jiangnan Univ	13	49	3.77	10	Islamic Azad Univ	32	32	1.00

**FIGURE 3:**
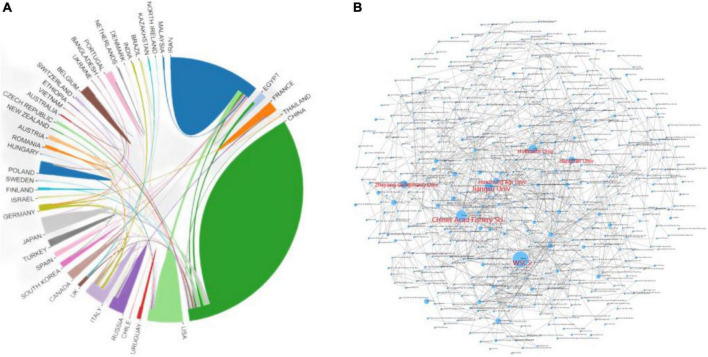
Cooperation between national/regional research institutions of sturgeon deep processing research in the world from 2010 to 2022. **(A)** Cooperation between countries and regions. **(B)** Cooperation of research institutions.

By analyzing the sending countries and institutions, we can show the distribution, reserves and cooperation of scientific research forces in sturgeon deep processing research. As shown on the left of Figure 3, countries cooperate closely in deep sturgeon processing; cross-country cooperation mainly focuses on the leading countries, and other countries are the nodes. China, Iran and the United States are not only at the forefront of sturgeon deep processing research but also maintain close cooperation with each other. In addition to Iran and the United States, the cooperation network with China as the leading country includes 14 countries, including Britain, Italy, Canada and Japan. The cooperation network with Iran as the main country includes eight countries, such as Italy, Canada, Spain, and France, in addition to China and the United States. The cooperation network with the United States as the main country only has cooperation relations with Italy, Canada, South Korea, Germany, Poland and Portugal. China has made outstanding contributions to global sturgeon deep processing. As seen from the institutional cooperation relationship on the right of Figure 3, the institutions at the cooperation nodes are the World Sturgeon Conservation Society (wscs), Chinese Acad fishery SCI, Jiangsu Univ, Huazhong Agri Univ, Hokkaido Univ, Zhejiang Gongshang Univ, and Jiangnan Univ. Although the world sturgeon conservation society has a considerable weight, there is no research cooperation relationship with various institutions. Cooperation links exist between the Chinese Academy of Fishery Sciences and two crucial research institutions, Jiangsu University and Huazhong Agricultural University. There are many cooperation nodes centered on this research institution. Huazhong Agricultural University cooperates with two important research institutions, the Chinese Academy of Fishery Sciences and Hokkaido University. There are eight research institutions centered on it. Hokkaido University has cooperative relations with Zhejiang Industrial and Commercial University and Huazhong Agricultural University; Zhejiang industry and Commerce have relations with Hokkaido and Dalian University of Technology, but there are few research cooperation institutions centered on them. Jiangsu University cooperates with the Chinese Academy of Fishery Sciences, but more than a dozen other research institutions are centered on it. Jiangnan University has less cooperation with domestic institutions, mainly with 12 international research institutions, such as Urmia Univ and Islamic Azad Univ. From the analysis of research institutions, it can be seen that in the research of sturgeon deep processing, one research institution is the center, and other research institutions focus on it. However, there is no deep cooperation between several major research institutions.

### Analysis of published periodicals

**FIGURE 4:**
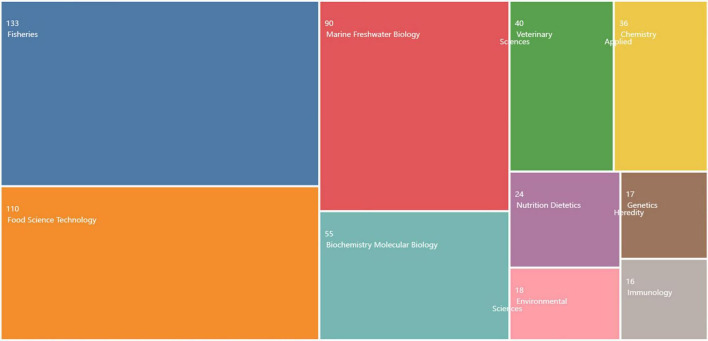
Global journal of sturgeon deep processing from 2010 to 2022.

According to the statistical findings of the industry fields of the selected documents in the WOS database (Figure 4), it can be seen that the research direction is in the three major research fields of fisheries, food science and technology, and marine and freshwater organisms. As shown in Table 2, through the analysis of the journals published by the selected literature through the online analysis platform of literature metrology, it is found that the top three journals are *JOURNAL OF APPLIED* ICHTHYOLOGY, *AQUACULTURE*, and *FOOD CHEMISTRY*. It is not difficult to see from the top ten journals in the number of publications that most of these journals are SCI area journals and top journals, among which FOOD CHEMISTRY and FOOD CONTROL are the authoritative journals in the food field. This shows that the academic influence of sturgeon deep processing has been influenced by authoritative national journals. However, the *JOURNAL OF APPLIED ICHTHYOLOGY*, which has the most papers published and the most frequently cited, is not a top journal. The Chinese Academy of Sciencess divides it into four areas regardless of major categories and subcategories. The appearance of this phenomenon also indicates the lack of research on the deep processing of sturgeon.

**TABLE 2:** Journal statistics of articles published from 2010 to 2022.

Journal name	Total number of articles	Total cited times	Average cited times
Journal of applied ichthyology	41	136	3.32
Aquaculture	16	7	0.44
Food chemistry	13	37	2.85
Food control	11	32	2.91
Fish & shellfish immunology	10	14	1.40
International journal of biological macromolecules	9	1	0.11
Aquaculture nutrition	9	21	2.33
Iranian journal of fisheries sciences	9	8	0.89
Fish physiology and biochemistry	7	14	2.00
Journal of the science of food and agriculture	6	20	3.33

### Analysis of research hotspots

**FIGURE 5:**
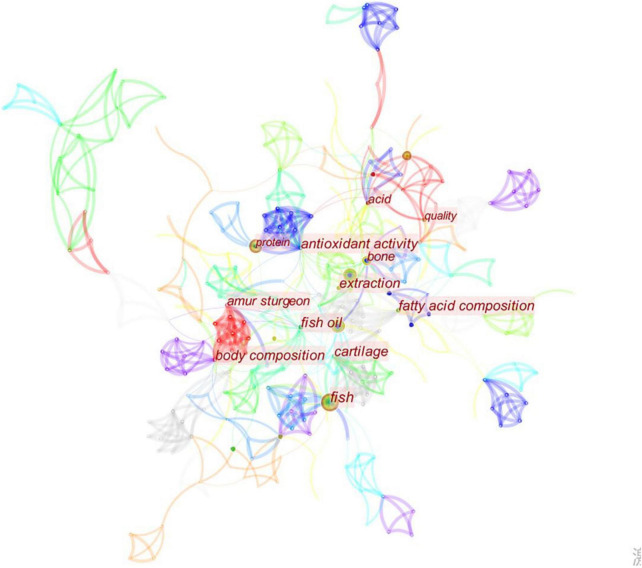
Keyword node map of deep sturgeon processing.

Research hotspots are the focus that scholars pay attention to and discuss at a particular stage in a specific field. Keywords are the refinement and generalization of the content expressed in an article, so they are often used to analyze the hot spots in a particular field. This paper visually analyzed the keywords of sturgeon processing research in WOS. A total of 315 nodes and 895 lines are obtained from the visualization map. Figure 5 shows that from 2010 to 2022, the high-frequency keywords for sturgeon deep processing research included “quality,” “fish oil,” “acid,” “protein,” and “active peptide,” in addition to the keyword “fish.” This shows that sturgeon research is not limited to preservation, and research on the deep processing of sturgeon and sturgeon byproducts has gradually deepened, which is of great significance to the development of the sturgeon industry in China.

**FIGURE 6:**
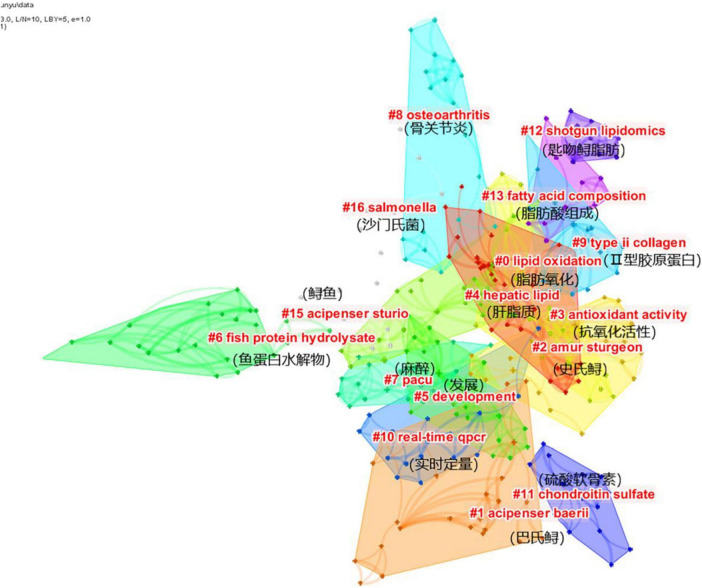
Clustering map of deep sturgeon processing.

Figure 6 shows the cluster analysis of sturgeon deep processing keywords in the WOS database. Keyword cluster analysis can further explain the internal relationship between keywords. Through cluster calculation, q = 0.8203 > 0.3 and s = 0.9328 > 0.7 show that clustering is effective. Among the 16 clustering modules, the modules related to sturgeon and sturgeon names were removed. According to the rank of clustering number, they were lipid oxidation, Acipenser schrenckii, antioxidant activity, liver lipid, development, fish protein hydrolysate, etc.

**FIGURE 7:**
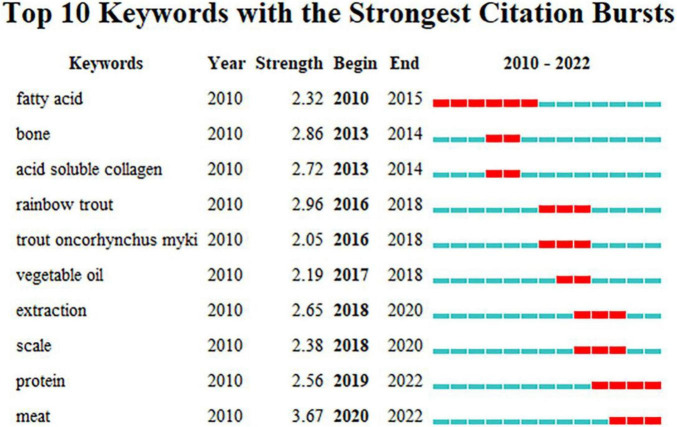
Keyword emergence map.

Figure 7 shows the emergence analysis of the keywords of sturgeon deep processing research in the WOS database. According to the number of papers issued by various countries, the fluctuation of sturgeon keywords may be more caused by the change in research direction in China. From 2010 to 2015, the key word fatty acid began to emerge. This phenomenon may be caused by the symposium on dietary fatty acid health held in Beijing in 2010. At this meeting, Professor William Sears of the University of California pointed out that ω-3 EPA/DHA can promote heart health and brain development and improve human immunity, but according to research, it is found that Chinese people ω-3. The intake of long-chain polyunsaturated fatty acids is far from the recommended value recommended by many authorities, which is a serious lack. Professor Su Yixiang of Sun Yat-sen University showed that α- in the three-month intervention experiment with linolenic acid and fish oil rich in EPA and DHA, it was found that EPA and DHA were comparable. α-Linolenic acid has a better effect on regulating blood lipids. Chinese consumers supplement DHA and EPA by using edible blended oil added to deep-sea fish oil in three meals a day, which is a convenient and appropriate method (38). At the same time, as the international market price of sturgeon caviar is generally more than 800 dollars per kilogram, China has set off a boom in sturgeon breeding (39, 40). In 2013, the Blue Book on Prevention of Osteoporosis issued by China led to research on the prevention of osteoporosis, among which sturgeon in large-scale cultivation has become a good research object. Since 2018, the keyword protein has become prominent, which may be because the research content of the three Nobel laureates in chemistry in 2018 is protein, thus setting off a research boom on protein. However, sturgeon chondroitin sulfate, which has been widely mentioned, has not been shown on the keyword highlighting map. This phenomenon may be due to the lack of research in this area, which requires further research.

## Development and research status of sturgeon byproducts

### Sturgeon skin/scales

Sturgeons are cartilaginous and hard phosphorous fish. The quantity of sturgeon skin accounts for 5–7% of the total fresh fish weight. This kind of skin is difficult to eat directly. In China, approximately 700 T or more of sturgeon skin is discarded annually. How to comprehensively utilize sturgeon processing wastes and control and reduce sturgeon processing costs has attracted increasing attention in the industry, which is also an urgent problem to be solved in the sturgeon processing industry (41). Yin Jian et al. (42) used sturgeon skin collagen as the primary material based on a single factor test, further optimized the preparation process conditions of dpp-IV inhibitory peptide in sturgeon skin by using response surface methodology, and determined the optimal enzymatic hydrolysis conditions as follows: enzymatic hydrolysis temperature 50°C, solid-liquid ratio 1%, pH 6.12, enzyme dosage 10170.35 U/g, and enzymatic hydrolysis time 12.12 h. The amino acid composition of the dpp-IV inhibitory peptide was analyzed (Table 3), and Feng et al. (43) extracted gastric soluble collagen (PSC) from sturgeon skin by enzymatic hydrolysis, with a maximum yield of 86.69%, thus proving that it is feasible to produce PSC from sturgeon skin. Wangyafei et al. adopted the method of hot water extraction to develop sturgeon skin gel products. The best boiling conditions were as follows: boiling temperature 80°C, boiling material:liquid ratio 1:4, and boiling time 2H. The gel strength of sturgeon skin gel obtained under this condition was 168.16 g; Color l* is 19.3, a* is 8.71, b* is 2.25; The recovery rate of collagen in the glue solution is 32.63%, and the content of soluble solids is 4.90% (44). Li Luyuan et al. used sturgeon skin as a raw material to prepare collagen peptides by alkaline protease hydrolysis. They analyzed the basic components of sturgeon skin, optimized the enzymatic hydrolysis conditions and studied the antioxidant activity of the enzymatic hydrolysis products. The results showed that the optimum enzymatic hydrolysis conditions were pH 9, temperature 55°C, mass fraction of enzyme 3% and hydrolysis degree 22.0% when the enzymatic hydrolysis time was 5 h (45). Atef et al. extracted collagen from sturgeon skin using acid and enzyme methods. The acid-soluble and enzyme-soluble collagen yields were 9.98% and 9.08%, respectively (46). Nikoo et al. first treated it with 0.05 m glacial acetic acid and then extracted it at 50 or 70°C for 1–6 h. The yield of gelatin was 9.42–12.47% (wet weight).

**TABLE 3:** Extraction methods and characteristics of byproducts from sturgeon skin (42, 44, 46).

Product	Technology	Characteristic
Dipeptidyl peptidase IV inhibitory peptide	Protein enzymolysis	The amino acid composition of the peptide with DPP-IV inhibitory activity is Gly-Pro-Ser-Gly-Leu-Asn-Gly-Ala-Lys. and the IC50 value can reach 61.27 ± 1.16 μM
Sturgeon skin jelly	Hot water extraction	The gel strength was 168.16 g; Color L * is 19.3, a * is 8.71, b * 2.25;
Sturgeon skin collagen	Acid extraction and enzymatic extraction	The yield of ASC extracted by the acid method was 9.98%, and the yield of PSC extracted by the enzyme method was 9.08%

### Sturgeon’s bones

The bones of fish include cartilage and hard bone. The cartilage content of the sturgeon is relatively high. The cartilage content in the head, notochord and fin accounts for approximately 10% of the body weight. It is an easy and stable source of cartilage for chondroitin sulfate (CS) extraction. CS is an acidic glycosaminoglycan that widely exists in the cartilage tissue of higher animals. It has many physiological functions, such as anticoagulation, anti-inflammatory, anticancer, relieving arthritis, reducing blood lipids, and preventing vascular sclerosis; it is also effective in treating coronary heart disease, angina pectoris, myocardial hypoxia and other cardiovascular diseases (47). Zhu et al. extracted collagen from sturgeon cartilage and speculated that it might contain type II collagen. The study found that this collagen retains the natural and complete triple helix structure and has the potential to replace collagen from mammals (48). Hu Yi et al. (49) extracted collagen from sturgeon cartilage. Their research shows that the collagen extracted from sturgeon cartilage is type II collagen, which is also consistent with the research results of Zhu et al. (48). Zhengping’an et al. (50) used sturgeon cartilage as a raw material to extract type II collagen and optimized its process parameters. The optimal process parameters for cartilage extraction were obtained: pyrolysis temperature 120°C for 30 min, enzyme amount 8,000 U/g pro, enzymatic hydrolysis time 4 h, enzymatic hydrolysis pH 8, and enzymatic hydrolysis temperature 50°C. The degree of hydrolysis of type II collagen extracted from sturgeon cartilage with the above optimal parameters was 10.85%, and the protein content was 70.7%. The content of mucopolysaccharide chondroitin sulfate in the extract was 30.6% (Table 4). Wuruiyun et al. (51) studied the enzymatic hydrolysis conditions of sturgeon cartilage collagen peptide when the enzyme amount was 1.5 × 105 IU/g, the ratio of bone powder to water was 1:25, the enzymatic hydrolysis time was 5 h, the enzymatic hydrolysis temperature was 50°C, and the ratio of alkaline protease to neutral protease enzyme activity was 1:2. The yield of bone collagen peptide was 72.36 ± 2.33%. It was also found that sturgeon bone collagen polypeptide could increase the activity of Caspase-3 by activating the apoptotic protease caspase-1, increasing the enzyme activity to 149.9 IU/μg. It can promote cell apoptosis and inhibit cell proliferation. Jun et al. (52) extracted chondroitin sulfate from sturgeon cartilage by alkali extraction, enzymatic hydrolysis and alcohol precipitation, degraded the chondroitin sulfate samples by ordinary comminution and ultrafine comminution combined with irradiation, and then studied the chemical structure of chondroitin sulfate by ion chromatography, UV–Vis spectral scanning, infrared absorption spectroscopy and nuclear magnetic resonance spectroscopy. The results showed that the chondroitin sulfate treated by ordinary comminution and ultrafine and irradiation degradation showed the typical characteristics of 4-chondroitin sulfate and 6-chondroitin sulfate, and the ultrafine comminution and irradiation treatment enhanced the proton signals of acetylgalactose and glucose on chondroitin sulfate. Rao Danhua et al. (53) used the testis tissue of sturgeon oracle bone plates as raw material through a single-factor experiment. Furthermore, they used the response surface software box Behnken design model to determine that the optimal extraction process conditions for sturgeon protamine were a sulfuric acid concentration of 0.83 mol/L, a liquid material ratio of 6.2:1 ml/g, an extraction time of 0.5 h, an extraction temperature of 35°C, and extraction 4 times. Under the optimal process conditions, the average yield of protamine reached 11.28%, the purity was as high as 86.13%, and the molecular weight was approximately 10 KU. In addition, 0.1 g/ml protamine was used to inhibit *Staphylococcus aureus*, and the diameter of the inhibition zone was 15.75 mm.

**TABLE 4:** Extraction methods and characteristics of byproducts from sturgeon bone (50–53).

Product	Technology	Characteristic
Type II collagen in sturgeon cartilage	Pyrolysis enzymatic hydrolysis	The degree of hydrolysis of collagen is 10.85%, the content of protein is 70.7%, and the content of mucopolysaccharide chondroitin sulfate is 30.6%
Sturgeon bone collagen polypeptide	Complex enzymatic hydrolysis technology of alkaline protease and neutral protease	The yield of collagen peptide is (72.36 ± 2.33)%. The protein can promote apoptosis and inhibit cell proliferation
chondroitin sulfate	Alkali extraction enzymatic hydrolysis alcohol precipitation method	Acipenser chondroitin sulfate contains GlcA(β 1 → 3) GalNAc-4SO3 and GlcA(β 1 → 3) GalNAc-6SO3 repeating unit structure.
Sturgeon protamine	Sulfuric acid extraction	The average yield of protamine is 11.28%, the purity is 86.13%, the molecular weight is approximately 10 ku, and it has good antibacterial activity.

### Sturgeon viscera

As the leftover from sturgeon processing, sturgeon viscera will pollute the environment if directly discarded. Taking professional treatment will increase the processing cost. Therefore, the treatment of sturgeon viscera has always been challenging. Currently, some researchers have conducted research on sturgeon viscera to improve its comprehensive utilization value. Ding Bingwen et al. (54) isolated and purified liver ferritin from Siberian sturgeon liver. The results showed that with increasing heat treatment temperature (60–100°C), the solubility and iron content of ABLF decreased gradually, and the content of protein aggregates increased. Infrared spectrum analysis showed that in ABLF α-, the relative content of helices decreased gradually, and the relative content of irregular curls increased as a whole. The above phenomenon is pronounced in the range of 80–100°C, while the change is small in the range of 60–80°C, indicating that ABLF has good thermal stability at 60–80°C. The iron release rate of ABLF increased with increasing temperature (25–65°C). The storage stability of ABLF at 4°C was higher than that at 25°C (Table 5). Yang Zifan et al. used sturgeon swim bladder and grass carp swim bladder as raw materials to compare and analyze the physical and chemical properties of enzyme soluble collagen (PSC) of the two swim bladders and concluded that grass carp swim bladder PSC has better thermal stability than sturgeon swim bladder PSC. However, sturgeon swim bladder PSC has a higher yield than grass carp swim bladder PSC (55). Ovissipour et al. studied the extraction rate of sturgeon visceral protein with five different enzymes and found that Alcalase had the best effect, with the highest protein recovery rate of 83.64% (56).

**TABLE 5:** Extraction methods and characteristics of byproducts from internal organs of sturgeon (54–56).

Product	Technology	Characteristic
Sturgeon liver ferritin	Heat treatment method	Acipenser liver ferritin has good thermal stability at 60–80°C
Enzyme soluble collagen	Enzymatic extraction	Sturgeon PSC belongs to type I collagen with high contents of Ser, Met, lle, Leu, His, Arg
Persian sturgeon visceral protein	Enzymolysis	Alcalase can make the recovery rate of sturgeon visceral protein reach 83.64%

### Sturgeon eggs

Sturgeon eggs can be pickled and processed into caviar, which is rich in essential amino acids such as isoleucine, leucine, lysine, high unsaturated fatty acids (EPA, DHA), inorganic salts, vitamins A, B, and D, and trace elements such as calcium, copper, magnesium, iron and selenium. It enjoys the reputation of “black gold” and “black pearl” in the international market. It is widely respected in Europe, America and other developed countries and has high economic value (1, 57). China has also launched a series of studies on sturgeon caviar as the first producer of sturgeon caviar. Shi Yugang et al. developed a natural biological preservative and found that sturgeon caviar treated with it can maintain its efficacy for a long time (58). Huang Yanqing et al. analyzed the components of caviar from cultured sturgeon and found that the nutritional components of caviar from wild sturgeon were similar (57). Zhan Shili (59) found that when the amount of salt added was higher than 3.5% of the weight of fish seeds, it could effectively prevent the growth and formation of *Clostridium botulinum*.

### Sturgeon oil

Sturgeon oil is rich in highly unsaturated fatty acids, higher than most terrestrial animals and plants. The rich eicosapentaenoic acid (EPA) and docosahexaenoic acid (DHA) have high medical value and are effective for oil deficiency, enhancing immunity, brain and intelligence, and protecting the retina. Research shows that it contains ω-3 polyunsaturated fatty acids, which can help alleviate non-alcoholic fatty liver disease and improve intestinal imbalance caused by high fat (13, 14, 60, 61). The content of polyunsaturated fatty acids in sturgeon oil is much higher than that of other freshwater fish, and sturgeon is easy to breed and has large output. Therefore, many researchers have studied sturgeon oil in recent years. Haoshuxian et al. extracted sturgeon oil under an enzyme amount of 0.6%, hydrolysis time of 2 h, hydrolysis temperature of 40°C, and pH 7. They found that the content of unsaturated fatty acids extracted by this method was high, and the degree of unsaturated fatty acids was close to that of deep-sea fish Kala (61).

## Problems and prospects

According to statistics, in recent years, as people have gradually changed their research on sturgeon from the simplest preservation to research on the potential value of collagen, chondroitin sulfate, and sturgeon oil, we found that sturgeon is a kind of aquatic product with great development value. The nutrients in sturgeon are more similar to those in deep-water fish. The essential amino acids rich in sturgeon are higher than those in common carp, crucian carp, chicken, pork and other terrestrial animals. The fish oil, active peptide and chondroitin sulfate in sturgeon play a significant role in improving human health. The oil unsaturation of sturgeon is higher than that of freshwater fish, and the difficulty of sturgeon culture is far lower than that of deep-sea fish. In addition, chondroitin sulfate in sturgeon has great use in medical care. However, through statistical analysis, it was found that global research on the processing and extraction of nutrients in sturgeon is seriously insufficient. China has made great contributions to sturgeon processing. We believe that further development of sturgeon can make great contributions to food therapy and medical care.

## Author contributions

JP: conceptualization, design of the work, writing—review and editing, supervision, and funding acquisition. ZL: visualization, resources, and writing—review and editing. RC, JW, and HA: conception or design of the work, resources, and investigation. WJ: writing—review and editing and supervision. QW and AAE-A: data acquisition, formal analysis, conceptualization, and writing—review and editing. All authors contributed to the article and approved the submitted version.

## Footnotes

1
www.webofscience.com

2
https://bibliometric.com
